# The impact of multimorbidity among adults with cardiovascular diseases on healthcare costs in Indonesia: a multilevel analysis

**DOI:** 10.1186/s12889-024-18301-7

**Published:** 2024-03-15

**Authors:** Royasia Viki Ramadani, Mikael Svensson, Sven Hassler, Budi Hidayat, Nawi Ng

**Affiliations:** 1 School of Public Health and Community Medicine, Institute of Medicine, Sahlgrenska Academy, University of Gothenburg, Gothenburg, Sweden; 2 Center for Health Economics and Policy Studies, Faculty of Public Health, Universitas Indonesia, Kota Depok, Indonesia; 3 Department of Pharmaceutical Outcomes & Policy, College of Pharmacy, University of Florida, Gainesville, USA; 4 Department of Health Sciences, Karlstad University, Karlstad, Sweden

**Keywords:** Non-communicable diseases, Multimorbidity, Hierarchical analysis, Health economics, Health insurance

## Abstract

**Background:**

Cardiovascular diseases (CVDs) are the leading cause of death in Indonesia, accounting for 38% of the total mortality in 2019. Moreover, healthcare spending on CVDs has been at the top of the spending under the National Health Insurance (NHI) implementation. This study analyzed the association between the presence of CVDs with or without other chronic disease comorbidities and healthcare costs among adults (> 30 years old) and if the association differed between NHI members in the subsidized group (poorer) and non-subsidized households group (better-off) in Indonesia.

**Methods:**

This retrospective cohort study analyzed the NHI database from 2016–2018 for individuals with chronic diseases (*n* = 271,065) ascertained based on ICD-10 codes. The outcome was measured as healthcare costs in USD value for 2018. We employed a three-level multilevel linear regression, with individuals at the first level, households at the second level, and districts at the third level. The outcome of healthcare costs was transformed with an inverse hyperbolic sine to account for observations with zero costs and skewed data. We conducted a cross-level interaction analysis to analyze if the association between individuals with different diagnosis groups and healthcare costs differed between those who lived in subsidized and non-subsidized households.

**Results:**

The mean healthcare out- and inpatient costs were higher among patients diagnosed with CVDs and multimorbidity than patients with other diagnosis groups. The predicted mean outpatient costs for patients with CVDs and multimorbidity were more than double compared to those with CVDs but no comorbidity (USD 119.5 vs USD 49.1, respectively for non-subsidized households and USD 79.9 vs USD 36.7, respectively for subsidized households). The NHI household subsidy status modified relationship between group of diagnosis and healthcare costs which indicated a weaker effect in the subsidized household group (β = -0.24, 95% CI -0.29, -0.19 for outpatient costs in patients with CVDs and multimorbidity). At the household level, higher out- and inpatient costs were associated with the number of household members with multimorbidity. At the district level, higher healthcare costs was associated with the availability of primary healthcare centres.

**Conclusions:**

CVDs and multimorbidity are associated with higher healthcare costs, and the association is stronger in non-subsidized NHI households. Households' subsidy status can be construed as indirect socioeconomic inequality that hampers access to healthcare facilities. Efforts to combat cardiovascular diseases (CVDs) and multimorbidity should consider their distinct impacts on subsidized households. The effort includes affirmative action on non-communicable disease (NCD) management programs that target subsidized households from the early stage of the disease.

**Supplementary Information:**

The online version contains supplementary material available at 10.1186/s12889-024-18301-7.

## Introduction

Cardiovascular diseases (CVDs) are the number one cause of death globally, taking an estimated 17.9 million lives in 2020, equivalent to 32% of all deaths worldwide [1]. In Indonesia, CVDs represented 38% of the total mortality in 2019 [2, 3], with stroke and ischemic heart diseases as the top two leading causes of death, accounting for 19% and 14% of all deaths, respectively [2]. The increasing prevalence of Non-Communicable Diseases (NCDs) also leads to increased healthcare spending on NCDs, with a total amount of USD 4,078 million or 22% of total healthcare spending in 2019, whereas CVDs accounted for 24% of the total NCDs healthcare spending [4]. About one-third of the Indonesian older population lives with multimorbidity [5], and more than 43% of National Health Insurance (NHI) users attending hospitals were identified with chronic multimorbidity [6]. The most common multimorbidity is hypertension with either diabetes mellitus, cerebral ischemia/chronic stroke, or ischemic heart disease [6].

Indonesia’s health system is decentralized, with a mixed system of public and private healthcare providers and financing [7]. The Government of Indonesia introduced the National Health Insurance (NHI) Scheme (*Jaminan Kesehatan Nasional/JKN*) in 2014. In 2023, the NHI scheme covered about 95% of the Indonesian population [8]; the remaining 5% population not covered by NHI included those working in informal sectors and unable to pay NHI contributions [9]. NHI is a unification of four existing health insurance programs: (1) *Jamkesmas,* the government-financed health insurance program targeting the poor and near-poor population; (2) *Askes,* the health insurance scheme for civil servants and pensioners; (3) *Jamsostek,* the insurance scheme for formal sector workers; and (4) *Jamkesda,* the local government budget-funded health insurance. Formal employees and civil servants pay the NHI contribution with 5% of their monthly salary, with 4% paid by the employers and 1% paid by employees. Informal sector workers contribute to the NHI by a fixed monthly premium (ranging from USD 2.9 to USD 10.4 per month). At the same time, the government subsidizes poor and near-poor individuals (NHI subsidized members) from general taxes with an amount of USD 2.9 per month.

The NHI has contracted 23,535 primary healthcare providers, 43% of which are public, and 2,616 hospitals, 52% of which are private [10]. NHI members have the option to visit both public and private providers. The primary healthcare providers serve as gatekeepers, referring patients to hospitals when necessary. Hospitals in Indonesia get reimbursed by the NHI based on diagnoses through Diagnosis Related Groups, while payment arrangements for Primary Healthcare centres are capitation. The NHI covers all medical costs for treatment in primary healthcare centers and hospitals without cost-sharing policies [11].

The introduction of NHI has led to an increase in healthcare utilization and improved access to out- and inpatient services. The proportion of individuals who received at least one outpatient treatment in the last 4 weeks has increased from 13.2% to 17.4% among the subsidized population and from 14.4% to 23.4% among the non-subsidized population when comparing the proportion before and after the NHI reform [12]. Previous studies show that the NHI expansion reduced healthcare access inequality across socioeconomic groups especially at private healthcare providers [12–14]. However, NHI was also found to favour populations living in urban areas and the better-off groups [15].

Previous population-based study have shown that patients with NCDs with multimorbidity (vs. without multimorbidity) have higher healthcare utilization in Indonesia [16]. However, at the same time, a population-based study conducted in 2018 suggested that nearly 70% of the respondents with a risk for CVDs failed to receive CVD treatments [17]. Lack of treatment for persons with CVDs increases long-term health risks, thus affecting individual long-term care conditions and increasing the economic burden for the patients, their families, and society [18]. As CVDs are among the most common chronic and disabling health problems in Indonesia and the world, it is essential to understand their impacts on healthcare spending. This study analyzed the association between the presence of CVDs with or without other chronic disease comorbidities and healthcare spending among adults aged 30 and over in Indonesia and whether the association differed between NHI- subsidized and non-subsidized households.

## Methods

### Study Population

This retrospective study was based on Indonesia's National Health Insurance (NHI) database. The NHI database was established in 2014 following the NHI Programme implementation, which now covers 95% of the Indonesian population, providing care to about 267 million people (2023) [8]. The NHI database includes membership, financial, and healthcare utilization data [19]. The healthcare utilization database provides data on primary diagnosis, treatments, drugs, costs, and basic social demographics such as age and gender.

### Sampling method, inclusion and exclusion criteria

**Fig. 1:**
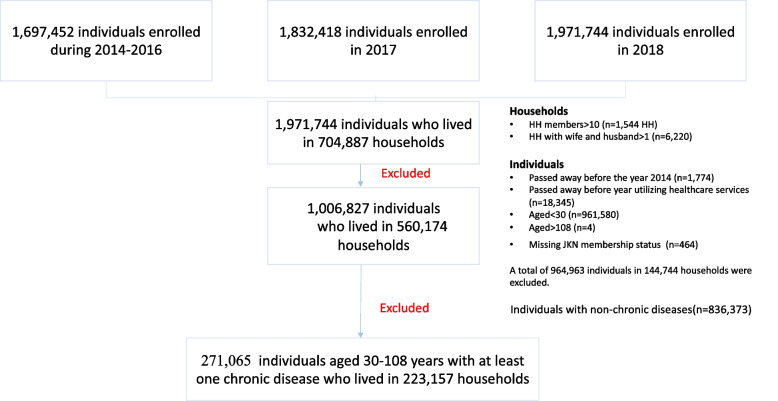
Study population: national health insurance data 2016–2018. The sample dataset consists a stratified sampling from members of the National Health Insurance enrolled in 2016 or earlier, 2017, and 2018. This study focus on individuals with chronic disease where number of individuals with chronic diseases between 2016–2018: 271,065 individuals with at least one chronic diseases who lived in 223,157 households

The NHI sample dataset was randomly stratified and sampled from members of the NHI enrolled in 2016 or earlier and new members in 2017 and 2018, forming a dynamic cohort (Fig. 1). Individuals selected from each cohort were included in the sample datasets. The sample dataset consisted of 1% of the total 73,441,160 households enrolled at 22,024 primary healthcare centres across 514 districts in Indonesia in 2016–2018, which summed up to 1,971,744, individuals who lived in 704,887 households [20].

We excluded households with more than 10 members (*n* = 1,544 HH) and households with more than one legally registered spouse which could be considered as data error (*n* = 6,220). Additionally, we excluded individuals who passed away during 2014 or earlier (*n* = 1,774), individuals who passed away before the year of utilizing healthcare services (*n* = 18,345), aged younger than 30 (*n* = 961,580) and over 108 years old (*n* = 4), and missing NHI membership subsidy status (*n* = 464). We excluded a total of 964,963 individuals who lived in 144,744 households. The final database from the dynamic cohorts consisted of 1,006,827 individuals who lived in 560,174 households between 2016 and 2018 (Fig. 1). Since this study focused on individuals with chronic diseases, we only included 271,065 individuals aged 30–108 years old with at least one chronic disease who lived in 223,157 households. More detailed sampling methods and selection criteria are explained in the Online Supplementary Document Sect. 1.

### Measurements

CVDs were defined based on the ICD-10 diagnosis (Table S1) in the Online Supplementary Document Sect. 2. Individuals who were recorded with CVDs and chronic diseases in an earlier year but did not have any actual visits in a later year are still classified as having chronic diseases. This study focused on chronic diseases [21] based on the ICD-10 classification [22]. Multimorbidity was defined as the presence of two or more chronic conditions [23] other than CVDs. Seventy-six diagnoses from ten families of diagnoses were used to generate multimorbidity status.

The primary outcome was healthcare costs for out- and inpatient visits associated with chronic diseases. Costs for out- and inpatient visits were presented in the USD value for 2018 (1 USD = IDR 14,481) [24]. The annual per-patient in- and outpatient costs were calculated as the sum of costs originated from all visits during the year based on tariffs paid using the Indonesian Case Based Groups payment scheme at the hospital and the non-capitation/Fee for Services scheme at primary healthcare centres [25].

We defined five groups of NHI members based on the presence of CVDs and multimorbidity of chronic diseases: (1) NHI members with no CVDs but with single chronic morbidity, (2) NHI members with no CVDs, but with multimorbidity, (3) NHI members with CVDs, but no comorbidity, (4) NHI members with CVDs and one comorbidity, and (5) NHI members with CVDs and multimorbidity.

We included individual, household, and district-level variables as covariates. At the individual level, we controlled for sex, age, marital status, types of primary healthcare centres registered, and region. Age was categorized into six groups (30–39, 40–49, 50–59, 60–69, 70–79 and 80 and above). Sex and marital status was a categorical variable. We divided Indonesian provinces into five regions, as presented in the Online Supplementary Documents Sect. 3 [25].

At the household level, we controlled for NHI household subsidy status, the total number of household members aged ≥ 30 years, and the proportion of household members with multimorbidity. The household subsidy status was constructed from the individual NHI-membership segmentations: NHI subsidized households constructed from government-subsidized members and non-subsidized households constructed from NHI members who are formal, retired, or informal workers. Households with both subsidized and non-subsidized members were categorized as subsidized households. The proportion of household members with multimorbidity was calculated as the number of household members with multimorbidity conditions compared to the total number of household members.

The district-level covariates included: (i) the density of healthcare facilities defined as the number of primary healthcare centres and hospitals per 10,000 NHI population, (ii) the proportion of NHI members who used NHI services, and (iii) the fiscal capacity index, defined by the Ministry of Finance, measures the district's capacity to raise revenues, either based on district revenues, transfer revenues from central funds, and other legitimate revenues at the district level [26–28]. The fiscal capacity index was calculated by dividing the district fiscal capacity by the national average of all district fiscal capacities. Very low fiscal capacity index < 0.548, low: 0.548–0.770, medium: 0.770–1.137, high: 1.137–2.021 and extremely high: >  = 2.021 [26]. Based on their fiscal capacity index, districts were classified into four categories: low (very low and low), medium, high and very high.

### Statistical analyses

A three-level linear mixed model was used to analyze the association between CVDs with or without other chronic disease comorbidities and healthcare costs accounted for covariates measured at the individual-, household-, and district levels. The cost outcome was initially transformed from USD using an inverse hyperbolic sine transformation (sinh ^−1^, IHS) for accounting for the many zeros and skewed cost data [27–31]. We conducted a cross-level interaction analysis to analyze if the association between CVDs with or without other chronic disease comorbidities and healthcare costs differed between NHI in subsidized (poorer) and non-subsidized households (better-off). The β coefficient for the interaction term was interpreted as the difference in the effect of different diagnoses on healthcare costs for NHI subsidized vs non-subsidized households. We retransformed the β coefficient to USD using cost value (x) = (exp^2x−1^)/(2exp^x^).

The intra-class correlation coefficient was used to estimate the residual variability at household and district levels. Multilevel analyses were conducted using unweighted data, as our analysis emphasizes tests of association and random effects rather than deriving nationally representative estimates. Analysis was performed using Stata/SE 17 (Stata Corp, College Station, TX, USA).

## Results

### Population characteristics

The proportion of patients diagnosed with CVDs and multimorbidity were lower in NHI subsidized households(1.7%) than in non-subsidized households (4.4%) (Table 1). Conversely, the proportion of patients with No CVDs with single chronic morbidity was higher in the subsidized households (79.2%) than in non-subsidized households (70.1%). At the household level, the proportion of household members with multimorbidity is 25.1% for NHI subsidized households and 31.6% for NHI non-subsidized households.

**Table 1:** Characteristics of the study participants at the baseline year based on households subsidized status

Variables	Households subsidy status		Total
	**Non-subsidized**	**Subsidized**	
**Individual level**			
**Overall**	152,296 (56.2)	118,769 (43.8)	271,065 (100.0)
**Disease groups**			
(1) No CVDs, but with single chronic morbidity	106,686 (70.1)	94,105 (79.2)	200,791 (74.1)
(2) No CVDs, but with multimorbidity	20,264 (13.3)	12,321 (10.4)	32,585 (12.0)
(3) CVDs, but no comorbidity	10,548 (6.9)	6,690 (5.6)	17,238 (6.4)
(4) CVDs and one comorbidity	8,084 (5.3)	3,698 (3.1)	11,782 (4.4)
(5) CVDs and multimorbidity	6,714 (4.4)	1,955 (1.7)	8,669 (3.2)
**Sex**			
Men	66,805 (43.9)	46,302 (39.0)	113,107 (41.7)
Women	85,491 (56.1)	72,467 (61.0)	157,958 (58.3)
**Age group**			
30–39	38,583 (25.3)	22,740 (19.2)	61,323 (22.6)
40–49	36,916 (24.2)	29,136 (24.5)	66,052 (24.4)
50–59	37,771 (24.8)	29,582 (24.9)	67,353 (24.8)
60–69	22,558 (14.8)	19,611 (16.5)	42,169 (15.6)
70–79	12,409 (8.2)	13,256 (11.2)	25,665 (9.5)
> = 80	4,059 (2.7)	4,444 (3.7)	8,503 (3.1)
**Marital status**			
Not married	11,698 (7.7)	8,589 (7.2)	20,287 (7.5)
Married	128,145 (84.1)	31,235 (26.3)	159,380 (58.8)
Divorced	12,078 (7.9)	3,628 (3.1)	15,706 (5.8)
Undefined	375 (0.3)	75,317 (63.4)	75,692 (27.9)
**Region**			
Region 1	92,044 (60.4)	73,879 (62.2)	165,923 (61.2)
Region 2	20,174 (13.3)	15,198 (12.8)	35,372 (13.0)
Region 3	28,931 (19.0)	24,066 (20.3)	52,997 (19.6)
Region 4	7,016 (4.6)	2,806 (2.3)	9,822 (3.6)
Region 5	4,131 (2.7)	2,820 (2.4)	6,951 (2.6)
**Household level**			
**Overall**	152,623 (68.4)	70,534 (34.6)	223,157 (100.0)
Proportions (%) of household members with multimorbidity (SD)	21.96 (31.64)	14.83 (25.19)	21.96 (31.64)
Mean total household number (SD)	1.94 (0.59)	1.48 (0.25)	2.21 (0.87)

Data is presented as N (number of observations), mean (SD) and the proportion (%) based on the households category (Non-subsidized vs subsidized). The baseline year is the year when the participants first enrolled (first diagnosed in the dataset) in the cohort. CVDs—cardiovascular diseases. All analyses were weighted with analytical sample weight

### Healthcare costs in five groups of chronic diseases

The mean healthcare costs were higher among patients diagnosed with CVDs and multimorbidity in out- and inpatient costs than in other groups (Table 2). The mean outpatient costs for patients with CVDs and multimorbidity were more than three times higher than those with no CVDs but with single chronic morbidity (USD 415.2 vs USD 121.8, respectively). Correspondingly, the average inpatient costs for patients with CVDs and multimorbidity were more than twice as much as those with no CVD but with single chronic morbidity (USD 1,135.2 vs USD 441.6, respectively). The mean costs of out- and inpatient visits were lower in patients in the subsidized group than in the non-subsidized group for all diagnosis groups.

**Table 2:** Average of annual outpatient and inpatient costs related to the presence of CVDs with or without other chronic disease comorbidities in US dollars between 2016 and 2018

Variable	Outpatient costs in USD Mean (SD)			Inpatient costs in USD Mean (SD)		
	**Overall (*N* = 215,660)**	**Individuals who belonged to non-subsidized households (*N* = 173,174)**	**Individuals who belonged to subsidized households (*N* = 42,486)**	**Overall (*N* = 114,918)**	**Individuals who belonged to non -subsidized households** **(*N* = 84,214)**	**Individuals who belonged to subsidized households** **(*N* = 30,704)**
**Overall**	179.6 (703.5)	195.81 (749.12)	137.0 (563.0)	634.8 (940.9)	720.3 (1056.3)	470.9 (634.7)
**Groups of patients**						
(1) No CVDs, but with single chronic morbidity	121.8 (500.9)	126.8 ( 523.1)	110.0 (444.6)	441.6 (508.9)	480.9 (553.4)	377.1 (418.2)
(2) No CVDs, but with multimorbidity	256.6 (964.1)	238.5 (1041.3)	181.4 (689.0)	757.1 (945.9)	848.8 (1050.4)	565.6 (636.6)
(3) CVDs, but no comorbidity	107.1 (176.5)	117.9 (190.01)	79.8 (132.8)	610.7 (950.0)	717.9 (1124.12)	444.5 (544.9)
(4) CVDs and one comorbidity	214.2 (702.45)	225.9 (701.78)	176.3 (703.5)	750.2 (1162.3)	843.1 (1276.1)	534.0 (799.1)
(5) CVDs and multimorbidity	415.2 (1214.12)	438.8 (1238.5)	312.1 (1096.3)	1135.2 (1602.9)	1213.2 (1672.7)	858.8 (1289.0)

Costs associated with out- and inpatient visits were presented in the U.S. dollar (USD) value for 2018. In- and outpatient costs were the average total costs associated with chronic diagnosis per patient annually. All costs presented in the analysis used a payer’s perspective based on the tariff paid by the healthcare insurance agency [25]. CVDs—cardiovascular diseases. All analyses were weighted with analytical sample weight

### Outpatient and inpatient costs: a multilevel analysis

Our model showed higher predicted out- and inpatient costs for patients with CVDs and multimorbidity (Tables 3 and 4). On average, patients with CVDs and multimorbidity had USD 1.17 higher outpatient costs (β = 1.00, 95% CI 0.99,1.02) (Table 3, Model 3) and USD 0.66 higher inpatient costs than patients with no CVDs, but with single chronic morbidity (β = 0.62, 95% CI 0.61,0.64) (Table 4, Model 3). Conversely, patients in subsidized households had USD 0.08 lower outpatient costs (β = -0.08, 95% CI -0.11,-0.06) (Table 3, Model 3) and USD 0.21 lower inpatient costs (β = -0.21, 95% CI -0.23,-0.18) (Table 4, Model 3).

**Table 3:** Association of individual, household, and district level characteristics with outpatient healthcare costs related to the presence of CVDs with or without other chronic disease comorbidities

Variable	Outpatient costs					
	**Model 1 **^**a**^** coefficient** (*n* = 215,660)	**(95% CI)**	**Model 2 **^**b**^** coefficient** (*n* = 215,660)	**(95% CI)**	**Model 3 **^**c**^** coefficient** (*n* = 215,660)	**(95% CI)**
**Individual level**						
**Group**						
(1) No CVDs, but with single chronic morbidity	REF	REF	REF	REF	REF	REF
(2) No CVDs, but with multimorbidity	0.55***	(0.54, 0.56)	0.49***	(0.47, 0.51)	0.49***	(0.48,0.51)
(3) CVDs, but no comorbidity	0.21***	(0.18, 0.23)	0.17***	(0.14, 0.19)	0.17***	(0.14,0.19)
(4) CVDs and one comorbidity	0.56***	(0.53, 0.58)	0.49***	(0.47, 0.51)	0.49***	(0.47,0.51)
(5) CVDs and multimorbidity	1.07***	(1.05, 1.09)	1.00***	(0.98, 1.02)	1.00***	(0.99,1.02)
**Household type**						
Non-subsidized	REF	REF	REF	REF	REF	REF
Subsidized	-0.08***	(-0.11, -0.06)	-0.08***	(-0.11, -0.06)	-0.08***	(-0.11,-0.06)
Group*Household type						
(1)*Subsidized						
(2)*Subsidized	-0.16***	(-0.19, -0.13)	-0.16***	(-0.19, -0.13)	-0.16***	(-0.19,-0.13)
(3)*Subsidized	-0.14***	(-0.19, -0.09)	-0.14***	(-0.18, -0.09)	-0.14***	(-0.18,-0.09)
(4)*Subsidized	-0.17***	(-0.22, -0.12)	-0.16***	(-0.21, -0.12)	-0.16***	(-0.21,-0.12)
(5)*Subsidized	-0.24***	(-0.29, -0.19)	-0.24***	(-0.29, -0.19)	-0.24***	(-0.29,-0.19)
**Household level**						
Proportions of household members with multimorbidity	-	-	0.16***	(0.14, 0.18)	0.16***	(0.15, 0.19)
The mean number of household members	-	-	0.01***	(0.01, 0.02)	0.02***	(0.01, 0.03)
**District level**						
Proportion of primary care per 10,000	-	-	-	-	0.02**	(0.00, 0.03)
Proportion of hospitals per 10,000	-	-	-	-	0.06***	(0.02, 0.11)
% of NHI members who utilized healthcare	-	-	-	-	-0.01	(-0.15, 0.12)
Fiscal category						
Low					0.01	(-0.01, 0.02)
Middle	-	-	-	-	-0.01	(-0.02, 0.01)
High	-	-	-	-	-0.03	(-0.05, -0.01)
Very high	-	-	-	-		
Intercept	4.51	(4.47, 4.55)	4.45	(4.40, 4.49)	4.41	(4.34, 4.49)
District level’s variance	0.16	(0.15, 0.17)	0.16	(0.15, 0.17)	0.16	(0.15, 0.18)
Household level’s variance	0.69	(0.68, 0.69)	0.68	(0.68, 0.69)	0.69	(0.68, 0.69)
ICC (district level)	0.02	(0.01, 0.02)	0.02	(0.01, 0.02)	0.02	(0.01, 0.02)
ICC (household level)	0.35	(0.34, 0.35)	0.35	(0.34, 0.35)	0.35	(0.34, 0.35)
Likelihood ratio test (LR)	18,666.79		18,441.15		17,485.34	

All models were also adjusted for sex, age, marital status, type of primary health care centres registered, and regions

^a^ Model 1: Multilevel linear regression with transformed outcome, cross-level between individual and controlled for individual-level covariates

^b^ Model 2: Multilevel linear regression with transformed outcome and controlled for individual and household-level covariates

^c^ Model 3: Multilevel linear regression with transformed outcome and controlled for individual, household, and district-level covariates

Coefficient is transformed using an inversed hyperbolic sine transformation (sinh ^−1^, IHS). We retransformed β coefficients to U.S. dollar (USD) using cost value (x) = (exp^2x−1^)/(2exp^x^). P-values were statistically significant at 1 percent (***), 5 percent (**) or 10 percent (*)

**Table 4:** Association of individual, household, and district level characteristics with inpatient healthcare costs related to the presence of CVDs with or without other chronic disease comorbidities

Variable	Inpatient costs					
	**Model 1 **^**a**^** Coefficient** (*n* = 114,918)	**(95% CI)**	**Model 2 **^**b**^** Coefficient** (*n* = 114,918)	**(95% CI)**	**Model 3 **^**c**^** Coefficient** (*n* = 114,918)	**(95% CI)**
**Individual level**						
**Group**						
(1) No CVDs, but with single chronic morbidity	REF	REF	REF	REF	REF	REF
(2) No CVDs, but with multimorbidity	0.39***	(0.38, 0.40)	0.38***	(0.36, 0.39)	0.38***	(0.36, 0.39)
(3) CVDs, but no comorbidity	0.18***	(0.17, 0.20)	0.18***	(0.16, 0.19)	0.18***	(0.16, 0.20)
(4) CVDs and one comorbidity	0.35***	(0.33, 0.36)	0.33***	(0.31, 0.35)	0.33***	(0.31, 0.35)
(5) CVDs and multimorbidity	0.64***	(0.62, 0.65)	0.62***	(0.60, 0.64)	0.62***	(0.61, 0.64)
**Household type**						
Non-subsidized	REF	REF	REF	REF	REF	REF
Subsidized	-0.21***	(-0.23,-0.19)	-0.21***	(-0.23,-0,19)	-0.21***	(-0.23, -0.18)
Group*Household type						
(1)*Subsidized						
(2)*Subsidized	-0.08***	(-0.11, -0.05)	-0.08***	(-0.10, -0,05)	-0.07***	(-0.10, -0.05)
(3)*Subsidized	-0.09***	(-0.12, -0.06)	-0.09***	(-0.12, -0.06)	-0.09***	(-0.13, -0.06)
(4)*Subsidized	-0.12***	(-0.15, -0.08)	-0.12***	(-0.15, -0.08)	-0.12***	(-0.15, -0.08)
(5)*Subsidized	-0.12***	(-0.15, -0.07)	-0.12***	(-0.15, -0.08)	-0.11***	(-0.15, -0.07)
**Household level**						
Proportions of household members with multimorbidity	-	-	0.03***	(0.02, 0.05)	0.04***	(0.03, 0.05)
The mean number of household members	-	-	-0.01***	(-0.02,-0.04)	-0.01**	(-0.02, -0.003)
**District level**						
Proportion of primary care per 10,000	-	-	-	-	0.04***	(0.03, 0.05)
Proportion of hospitals per 10,000	-	-	-	-	-0.01	(-0.04, 0.02)
% of NHI members who utilized healthcare	-	-	-	-	-0.00	(-0.12, 0.11)
Fiscal category						
Low						
Middle	-	-	-	-	-0.03***	(-0.05, -0.02)
High	-	-	-	-	-0.07***	(-0.09, -0.06)
Very high	-	-	-	-	-0.11***	(-0.13, -0.08)
Intercept	6.70	(6.67, 6.73)	6.72	(6.68, 6.74)	6.72	(6.66, 6.78)
District level’s variance	0.09	(0.09, 0.11)	0.09	(0.09, 0.11)	0.11	(0.10, 0.12)
Household level’s variance	0.34	(0.33, 0.35)	0.34	(0.33, 0.35)	0.33	(0.33, 0.35)
ICC (district level)	0.02	(0.01, 0.02)	0.02	(0.01, 0.02)	0.02	(0.02, 0.03)
ICC (household level)	0.21	(0.19, 0.22)	0.21	(0.20, 0.22)	0.21	(0.20, 0.22)
Likelihood ratio test (LR)	2,741.55		2,726.31		2,713.98	

All models adjusted with sex, age, marital status, type of primary healthcare centres registered, and regions

^a^ Model 1 Multilevel linear regression with transform outcome, cross-level between individual and controls for individual-level covariates

^b^ Model 2 Multilevel linear regression with transform outcome and controls for individual and household covariates

^c^ Model3 Multilevel linear regression with transform outcome and controls for individual, household, and district covariates

Coefficient is transformed using an inversed hyperbolic sine transformation (sinh ^−1^, IHS). To retransform β coefficient to U.S. dollar (USD) cost value (x) = (exp^2x−1^)/(2exp^x^). P-value statistically significant at 1 percent (***), 5 percent (**) or 10 percent (*) of the confidence intervals

A significant effect modification of household subsidy status on healthcare costs indicated a weaker effect in the subsidized household for out- and inpatient costs compared to the non-subsidized group (Table 3, Model 3). The interaction was significant for all five diagnosis groups, meaning that the association of CVDs and multimorbidity on healthcare costs were modified by NHI household subsidy status both in out- and inpatient costs.

**Fig. 2:**
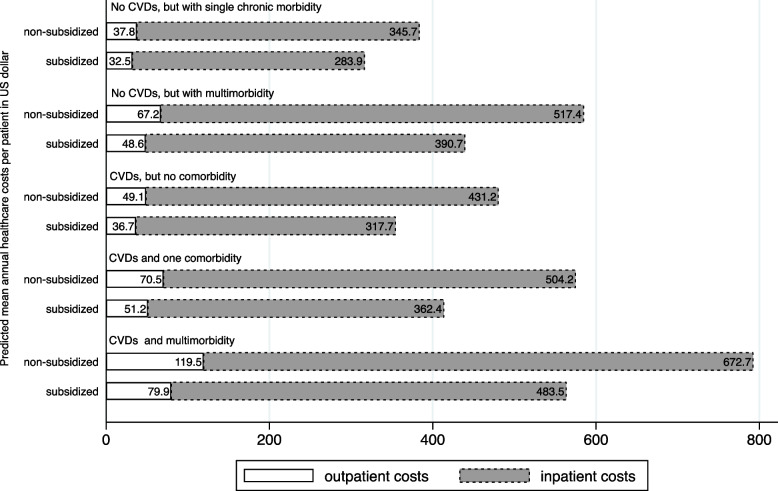
Predicted mean annual healthcare costs per patient by diagnosis group (2016–2018) in U.S. dollars (USD). Costs associated with out- and inpatient visits are presented in the USD value for 2018. Predicted costs were calculated from the transformed coefficient to USD cost value (x) = (exp^2x−1^)/(2exp^x^). Costs were predicted using three-level multilevel linear regression with transform outcome and controlled for individual, household, and district covariates

The modification of household subsidy status on the association between the group of diagnosis and healthcare costs is illustrated in Figure S2 and Figure S3 in Online Supplementary Document Sect. 4. Our final model showed that the highest predicted out- and inpatient costs were found for patients with CVDs and multimorbidity in NHI non-subsidized households (Fig. 2). Predicted mean outpatient costs for the CVDs and multimorbidity group were more than double the outpatient costs for those diagnosed with CVDs, but no comorbidity ( USD 119.5 vs USD 49.1, respectively for non-subsidized and USD 79.9 vs USD 36.7, respectively for subsidized households).

For inpatient costs, patients in the CVDs and multimorbidity groups also had higher mean costs than those with CVDs, but no comorbidity (USD 672.7 vs USD 431.2, respectively for the non-subsidized group and USD 483.5 vs USD 317.7, respectively for the subsidized group).

### Household and district effects on healthcare costs

In our final model, which controlled for individual, household, and district covariates (Tables 3 and Table 4, Model 3), we observed the Intraclass Correlation (ICC) for outpatient costs at the district and household levels was 2% and 35%, respectively, and 2% and 21% for inpatient costs, respectively. The ICC indicated that the household level accounted for 35% of the total variance in outpatient costs and 21% total variance in inpatient costs. At the same time, the district level accounted for 2% of the total variance in outpatient costs and inpatient costs.

Our results suggested that households with a one percent higher of household members with multimorbidity contributed to higher outpatient costs (USD 0.16) (β = 0.16, 95% CI 0.15,0.19) and inpatient costs (USD 0.04) (β = 0.04, 95% CI 0.03,0.05) (Tables 3 and Table 4, Model 3). At the same time, a higher number of household members contributed to higher outpatient costs by USD 0.04 (β = 0.04, 95% CI 0.03,0.05) but lower inpatient costs by USD 0.01(β = -0.01, 95% CI -0.02,-0.003). The availability of primary healthcare centres was positively associated with out- and inpatient costs. In addition, the availability of hospitals contributed to higher outpatient costs. Districts with higher fiscal capacity had lower inpatient costs than districts with low fiscal capacity.

Sensitivity analyses were performed assuming that households with both subsidized and non-subsidized members were categorized as non-subsidized households. The results presented in the Online supplementary document, Sect. 4 (Table S4-S7, and Figure S4) where the conclusions were similar to the main analysis: CVD and multimorbidity were associated with higher healthcare costs, and the association is more substantial in non-subsidized NHI households.

## Discussion

In the present study, we found that healthcare costs of individuals with chronic diseases is associated at the individual level with the existence of CVDs and or multimorbidity. At the household level, the healthcare costs is associated with the percentage of household members with multimorbidity, and the mean number of household members, and at the district-level with the proportion of hospitals, proportion of primary healthcare centre, and district’s fiscal capacity. These findings suggest that CVDs and multimorbidity are associated with higher healthcare costs compared to the other diagnosis groups, and the association was attenuated by being in subsidized households.

At the household level, the number of household members with multimorbidity is associated with higher out- and inpatient costs. At the same time, the number of household members in the households is associated with higher outpatient costs but lower inpatient costs. At the district level, the availability of primary healthcare centres is associated with higher out- and inpatient costs. In addition, the availability of hospitals is associated with higher outpatient costs. While being in a higher fiscal category district is associated with lower inpatient costs.

Our results are similar to a previous systematic review where multimorbidity was shown to impose a large economic burden on the health system and society, particularly with diabetes and heart/vascular conditions imposing large annual costs (International Dollar (I$) 37,090) [32]. Earlier studies in NHI settings, both in Indonesia and Vietnam, also show that Type 2 Diabetes Mellitus patients with complications had twice the costs compared to those without complications (USD 1,047 vs USD 431, respectively in Indonesia) [33] and (USD 398 vs USD 205, respectively in Vietnam) [34]. In addition, previous population-based studies in which patients with chronic diseases had higher odds of higher catastrophic healthcare costs are in line with our findings [16, 35]. This population-based study estimated total Out of Pocket Expenditure for out- and inpatient costs is USD 968 for individuals with at least 3 NCDs compared to USD 292 for individuals with 1 NCD [16].

The association of CVDs and multimorbidity and out- and inpatient costs implies higher care costs than the other diagnosis groups (patients with No-CVDs, but with single chronic morbidity, No CVDs but multimorbidity, CVDs but no comorbidity, CVDs and one comorbidity). This evidence strengthens the relevance for the government's policy to prioritize CVDs prevention, driven by the fact that CVDs are the primary cause of death in the country, and previously most of the government's public spending was spent on NCDs and CVDs treatments [4].

Our results showed that the lowest out- and inpatient costs were incurred among individuals in the NHI subsidized group. Those receiving subsidies have lower socioeconomic status and face more unmet needs for cardiovascular care [17]; hence may be confronted with less healthcare utilization and lower costs [12, 13]. Household subsidy status can be interpreted as an indirect socio-economic inequality that hinders access to health facilities. Previous studies also showed that even though the NHI program has reduced the magnitude of inequality in accessing care across different socioeconomic groups, access remains an issue among populations who belong to the lowest quintile*.* [12, 13]. Except for access to public primary healthcare, healthcare access remains pro-rich in Indonesia [13]. The probability of seeking outpatient and inpatient care is higher among the non-subsidized group than the subsidized group (by 7.9 percent vs 2 percent, respectively, for outpatient services and by 8.2 percent vs 1.7 percent for inpatient services) [12].

The variation of costs is profound beyond the individual level; households with a higher percentage of multimorbidity significantly predict higher outpatient costs. This finding is similar to previous findings in Bangladesh [36] and Tanzania [37], where households with chronic illnesses (NCDs and other long-run illnesses, including TB and HIV) are associated with higher healthcare financial burdens. The variation between households is persistent after controlling for household-level variables.

Unmeasured household factors that still exist after adjusting for household-level variables are possibly due to variables not included in the current analysis, such as knowledge and attitude towards health, behaviour, and norms perception, which affect healthcare utilization, which according to the household production of health places household at the centre of the process [38]. Therefore, the focus on tackling NCDs and multimorbidity should be directed towards the individual and household levels. The existing chronic disease management programme under NHI, called *PROLANIS* [39], which targets patients with chronic diseases, including diabetes and hypertension, should focus not only on the individual level but also on the household level, targeting subsidized households at an early stage.

This study found a small variation of out- and inpatient costs at the district level. This finding is similar to a previous multilevel study in Indonesia [16] on access to healthcare and child immunization [40]. A study on unmet need for CVDs also found that no determinant at the district level was significantly associated with the unmet needs of CVDs in Indonesia [17]. Our study showed a positive association between the availability of primary healthcare centres and out- and inpatient costs and the availability of hospitals with higher outpatient costs. This finding is similar to a previous study that reported a positive effect of healthcare facilities on healthcare utilization [41]. Further studies are required to understand what drives the variation in healthcare utilization at the district level. The characteristics of providers in Indonesia are not homogenous and vary significantly among healthcare providers [41].

Though this study used extensive and national-level administrative health insurance data, it has several limitations. The NHI data lacks integration with other administrative data, limiting the possibility of controlling for other potential socioeconomic confounding factors in the analysis. In addition, the NHI data only capture out-patient and in-patient costs at hospital and non-capitation costs at the primary healthcare centre. It does not capture reimbursement at primary healthcare which is based on capitation.

As this study is based on national claims data, the cost data and analytical results should be interpreted cautiously. The costs reported in this study were from the perspective of the payer (NHI agency). However, there are several limitations to estimating costs from claims-based data, such as potential underestimation or overestimation of payments [42, 43]. In addition, the quality and reliability of medical coding [42–44], which serves as the basis for diagnostic information, may vary between hospitals and could affect the cost estimation of a case-based group payment.

## Conclusions

CVDs and multimorbidity are associated with higher healthcare costs, and the association is stronger in non-subsidized households. Households' subsidized status can be construed as indirect socioeconomic inequality that hampers access to healthcare facilities. Efforts to control cardiovascular diseases (CVDs) and multimorbidity should consider their distinct impacts on subsidized households. This effort includes affirmative action on non-communicable disease (NCD) management programs that target subsidized households from the early stage.

### Supplementary Information

**Supplementary Material 1.**

## Data Availability

The data are available from National Social Security Agency for Health, but restrictions apply to the availability of these data, which were used under license for the current study, and so are not publicly available. Data are available from the corresponding author upon reasonable request and with permission of the National Social Security Agency for Health. The data were de-identified, and researchers interested in using this dataset can find details at https://data.bpjs-kesehatan.go.id. After submitting the manuscript, the NHI Agency publishes the newer dataset, which covers the period up to 2022 in December 2023.

## References

[1] World Health Organization. Cardiovascular Diseases. 2020. Available: https://www.who.int/health-topics/cardiovascular-diseases/#tab=tab_1. Accessed: 24 March.

[2] Institute for Health Metrics and Evaluation .Global Burden of Diseases: Indonesia. 2019. Available: https://www.healthdata.org/data-visualization/gbd-compare. Accessed.

[3] World Health Organization .World Health Organization-Noncommunicable Diseases (NCD) Country Profile - Indonesia. 2018. Available: https://www.who.int/nmh/countries/idn_en.pdf. Accessed: 16 October 2019.

[4] Ministry of Health. National Health Accounts Indonesia 2019. 2021. Available: https://ppjk.kemkes.go.id/libftp/uploads/trs_local_nposth/120/Report%20Full%20Figure%20NHA%202019_final_13012022.pdf. Accessed.

[5] Hussain MA, Huxley RR, Al MA (2015). Multimorbidity Prevalence and Pattern in Indonesian Adults: An Exploratory Study using National Survey Data. BMJ Open.

[6] Husnayain A, Ekadinata N, Sulistiawan D, Chia-Yu SuE (2020). Multimorbidity Patterns of Chronic Diseases among Indonesians: Insights from Indonesian National Health Insurance (INHI) Sample Data. Int J Environ Res Public Health.

[7] Mahendradhata Y, Trisnantoro L, Listyadewi S, Soewondo P, Marthias T, Harimurti P, et al. The Republic of Indonesia Health System Review. Health Systems in Transition, Vol-7 No.1 ed. New Delhi: WHO Regional Office for South-East Asia; 2017 2017.

[8] National Health Insurance Agency. Number of JKN Member (In Indonesian: BPJS Kesehatan. Peserta Program JKN). 2023. Available: https://faskes.bpjs-kesehatan.go.id/aplicares/#/app/peta. Accessed.

[9] Muttaqien M, Setiyaningsih H, Aristianti V, Coleman HLS, Hidayat MS, Dhanalvin E (2021). Why did informal sector workers stop paying for health insurance in Indonesia? Exploring enrollees' ability and willingness to pay. PLoS ONE.

[10] BPJS Kesehatan. NHI Health Facilities (In Bahasa Indonesia: “Fasilitas Kesehatan JKN”). 2023. Available: https://faskes.bpjs-kesehatan.go.id/aplicares/#/app/ketersediaanfaskes. Accessed.

[11] Agustina R, Dartanto T, Sitompul R, Susiloretni KA, Achadi EL, Taher A, et al. Universal health coverage in Indonesia: concept, progress, and challenges. The Lancet. 2018. 10.1016/S0140-6736(18)31647-7 30579611

[12] Erlangga D, Ali S (2019). Bloor KJIjoph. The impact of public health insurance on healthcare utilisation in Indonesia: evidence from panel data.

[13] Johar M, Soewondo P, Pujisubekti R, Satrio HK, Adji A (2018). Inequality in access to health care, health insurance and the role of supply factors. Soc Sci Med.

[14] Anindya K, Lee JT, McPake B, Wilopo SA, Millett C, Carvalho N. Impact of Indonesia’s national health insurance scheme on inequality in access to maternal health services: A propensity score matched analysis. Journal of global health. 2020;10. 10.7189/jogh.10.010429 PMC7298736 32566167

[15] Sambodo NP, Van Doorslaer E, Pradhan M, Sparrow R (2021). Does geographic spending variation exacerbate healthcare benefit inequality? A benefit incidence analysis for Indonesia. Health Policy Plan.

[16] Marthias T, Anindya K, Ng N, McPake B, Atun R, Arfyanto H (2021). Impact of non-communicable disease multimorbidity on health service use, catastrophic health expenditure and productivity loss in Indonesia: a population-based panel data analysis study. BMJ Open.

[17] Maharani A, Tampubolon G (2014). Unmet needs for cardiovascular care in Indonesia. PLoS ONE.

[18] Dunbar SB, Khavjou OA, Bakas T, Hunt G, Kirch RA, Leib AR (2018). Projected costs of informal caregiving for cardiovascular disease: 2015 to 2035: a policy statement from the American Heart Association. Circulation.

[19] Ng JYS, Ramadani RV, Hendrawan D, Duc DT, Kiet PHT. National Health Insurance Databases in Indonesia, Vietnam and the Philippines. PharmacoEconomics - open. 2019. 10.1007/s41669-019-0127-2 PMC6861401 30859490

[20] Ariawan I, Sartono B, Jaya C, Mawardi J, Sodiq J, Baros W, et al. National Health Insurance Sample Data year 2015–2016 (In Indonesian: Data Sampel BPJS Kesehatan Tahun 2015–2016). Jakarta: BPJS Kesehatan. 2019.

[21] Agency for Healthcare Research and Quality R, MD,. Tools Archive for the Chronic Condition Indicators for ICD-10-CM. Healthcare Cost and Utilization Project (HCUP). 2020. Available: https://www.hcup-us.ahrq.gov/toolssoftware/chronic_icd10/chronic_icd10.jsp#overview. Accessed.

[22] Institute for Health Metrics and Evaluation. Global Burden of Disease Study 2017 (GBD 2017) Causes of Death and Nonfatal Causes Mapped to ICD Codes. 2017. Available: http://ghdx.healthdata.org/record/ihme-data/gbd-2017-cause-icd-code-mappings. Accessed.

[23] Koller D, Schön G, Schäfer I, Glaeske G, van den Bussche H, Hansen H (2014). Multimorbidity and Long-term Care Dependency—a five-year follow-up. BMC Geriatr.

[24] Central Buerau Statistics of Indonesia. Indonesia Inflation by Expenditure Group (In Indonesian: Badan Pusat Statistik. Inflasi Indonesia Menurut Kelompok Pengeluaran). 2020. Available: https://www.bps.go.id/statictable/2009/06/29/901/inflasi-indonesia-menurut-kelompok-pengeluaran-2006-2019.html. Accessed: 23 August 2022.

[25] Ministry of Health Republic Indonesia. Regulation of the Minister of Health of the Republic of Indonesia No. 64 of 2016 concerning Health Service Tariff Standards in the Implementation of the Health Insurance Program (In Indonesian: Kementerian Kesehatan. Peraturan Menteri Kesehatan Republik Indonesia No 64 Tahun 2016 Tentang Standar Tarif Pelayanan Kesehatan dalam Penyelenggaraaan Program Jaminan Kesehatan). 2016.

[26] Regulation of the Minister of Finance of the Republic of Indonesia No 107/PMK.07/2018 on Regional Fiscal Capacity Map (In Indonesian: Kementerian Keuangan. Peraturan Menteri Keuangan Republik Indonesia No 107/PMK.07/2018 Tentang Peta Kapasitas Fiskal Daerah), (2018).

[27] Moghimbeigi A, Eshraghian MR, Mohammad K, Mcardle B (2008). Multilevel zero-inflated negative binomial regression modeling for over-dispersed count data with extra zeros. J Appl Stat.

[28] Manning WG, Basu A, Mullahy J (2005). Generalized modeling approaches to risk adjustment of skewed outcomes data. J Health Econ.

[29] Manning WG, Mullahy J (2001). Estimating log models: to transform or not to transform?. J Health Econ.

[30] Diehr P, Yanez D, Ash A, Hornbrook M, Lin D (1999). Methods for analyzing health care utilization and costs. Annu Rev Public Health.

[31] Burbidge JB, Magee L, Robb AL (1988). Alternative transformations to handle extreme values of the dependent variable. J Am Stat Assoc.

[32] Tran PB, Kazibwe J, Nikolaidis GF, Linnosmaa I, Rijken M, van Olmen J (2022). Costs of multimorbidity: a systematic review and meta-analyses. BMC Med.

[33] Hidayat B, Ramadani RV, Rudijanto A, Soewondo P, Suastika K, Siu Ng JY. Direct Medical Cost of Type 2 Diabetes Mellitus and Its Associated Complications in Indonesia. Value Health Reg Issues. 2022;28:82–9. 10.1016/j.vhri.2021.04.006 34839111

[34] Tuan Kiet Pham H, Tuyet Mai Kieu T, Duc Duong T, Dieu Van Nguyen K, Tran NQ, Hung Tran T, et al. Direct medical costs of diabetes and its complications in Vietnam: A national health insurance database study. Diabetes Res Clin Pract. 2020:108051. 10.1016/j.diabres.2020.108051 32027924

[35] Anindya K, Ng N, Atun R, Marthias T, Zhao Y, McPake B (2021). Effect of multimorbidity on utilisation and out-of-pocket expenditure in Indonesia: quantile regression analysis. BMC Health Serv Res.

[36] Rahman MM, Islam MR, Rahman MS, Hossain F, Alam A, Rahman MO (2022). Forgone healthcare and financial burden due to out-of-pocket payments in Bangladesh: a multilevel analysis. Health Econ Rev.

[37] Counts CJ, Skordis-Worrall J (2016). Recognizing the importance of chronic disease in driving healthcare expenditure in Tanzania: analysis of panel data from 1991 to 2010. Health Policy Plan.

[38] Berman P, Kendall C, Bhattacharyya K (1994). The household production of health: integrating social science perspectives on micro-level health determinants. Soc Sci Med.

[39] Rachmawati S, Prihhastuti-Puspitasari H, Zairina E (2019). The implementation of a chronic disease management program (Prolanis) in Indonesia: a literature review. J Basic Clin Physiol Pharmacol.

[40] Maharani A, Tampubolon G (2014). Has decentralisation affected child immunisation status in Indonesia?. Glob Health Action.

[41] Mulyanto J, Kunst AE, Kringos DS. The contribution of service density and proximity to geographical inequalities in health care utilisation in Indonesia: A nation-wide multilevel analysis. Journal of Global Health. 2020;10. 10.7189/jogh.10.020428 PMC7719271 33312501

[42] Supriadi S, Putri IA, editors. Overview of Claim Return for Outpatient JKN Hospital X in South Tangerang. Proceedings; 2023: MDPI.

[43] Samsulhadi A, Chalidyanto D (2020). Factors affecting potential overpayment claim of government health insurance in naval hospital. EurAsian Journal of BioSciences.

[44] Chalkley M, Hidayat B, Ramadani RV, Aragón MJ. The sensitivity of hospital coding to prices: evidence from Indonesia. International journal of health economics and management. 2021:1–16. 10.1007/s10754-021-09312-7 PMC9090886 34491464

