# Supplementary material for: The impact of multimorbidity among adults with cardiovascular diseases on healthcare costs in Indonesia: a multilevel analysis
Source: BMC Public Health. 2024 Mar 15;24:816. doi: 10.1186/s12889-024-18301-7 (PMC10941372; doi:10.1186/s12889-024-18301-7)
Supplement: Supplementary file 1 — Supplementary Material 1. [file 12889_2024_18301_MOESM1_ESM.docx]

# Online supplementary documents

## Section 1. Sampling method, inclusion and exclusion criteria

The NHI sample dataset was randomly stratified and sampled from members of the NHI enrolled in 2016 or earlier, 2017, and 2018, thereby forming three sub-populations. Individuals and households selected in the first sampling period in 2016 were also selected in 2017 and 2018. The sampling frame for the NHI sample dataset was listed from 73,441,160 families enrolled as members at 22,024 primary healthcare centers across 514 districts in the country. The latter is defined by their history of healthcare utilization, resulting in three different classifications: 1. Households that have never utilized healthcare services, 2. Households that have ever visited gatekeepers, and 3. Households that have visited primary and referral care. If all primary healthcare centers have three types of NHI households, there will be three strata in primary healthcare centre. Within each stratum, a total of 10 families were randomly sampled. All families became a sample in each stratum if the number of families within strata was < 10. If selected, all individuals in the family were included in the dataset

A sample of 586,969 households was subsequently selected, constituting 1,697,452 individuals. NHI members registered in 2017 and 2018 were added with a similar sampling strategy resulting in an additional 56,791 households with 134,966 individuals for 2017 (n=1,832,418 individuals) and 60,164 households with 139,326 individuals for 2018 (n=1,971,744 individuals). From an initial total of 1,971,744 individuals who lived in 704,887 households, we excluded households with more than 10 members (n=1,544 HH) and households with more than one legally registered spouse which could be considered as data error (n=6,220). Additionally, we excluded individuals who passed away during 2014 or earlier (n=1,774) and passed away before the year of utilizing healthcare services (n=18,345), aged younger than 30 (n=961,580) and over 108 years old (n=4), and missing NHI membership subsidy status (n=464). We excluded—a total of 964,963 individuals who lived in 144,744 households. The final database from the dynamic cohorts consisted of 1,006,827 individuals who lived in 560,174 households between 2016 and 2018 (Figure 1). Since this study focused on individuals with chronic diseases, we only included 271,065 individuals aged 30-108 years old with at least one chronic disease who lived in 223,157 households.

## Section 2. List of ICD-10 codes of non-communicable diseases

**Table S1.** List of ICD-10 codes of non-communicable diseases

| **No** | **Diagnosis (Cause)** | **ICD-10** |  |
| --- | --- | --- | --- |
| **Cardiovascular Diseases** | | | |
| 1 | Rheumatic heart disease | I01-I01.9, I02.0, I05-I09.9 | |
| 2 | Ischemic heart disease | I20-I25.9 | |
| 3 | Stroke | G45-G46.8, I60-I63.9, I65-I66.9, I67.0-I67.3, I67.5-I67.6, I68.1-I68.2, I69.0-I69.3 | |
| 4 | Ischemic stroke | G45-G46.8, I63-I63.9, I65-I66.9, I67.2-I67.3, I67.5-I67.6, I69.3 | |
| 5 | Intracerebral hemorrhage | I61-I62, I62.1-I62.9, I68.1-I68.2, I69.1-I69.2 | |
| 6 | Subarachnoid hemorrhage | I60-I60.9, I62.0, I67.0-I67.1, I69.0 | |
| 7 | Hypertensive heart disease | I11-I11.9 | |
| 8 | Non-rheumatic valvular heart disease | I34-I37.8 | |
| 9 | Non-rheumatic calcific aortic valve disease | I35-I35.9 | |
| 10 | Non-rheumatic degenerative mitral valve disease | I34-I34.9 | |
| 11 | Other non-rheumatic valve diseases | I36-I37.8 | |
| 12 | Cardiomyopathy and myocarditis | B33.2, I40-I41.9, I42.1-I42.8, I43-I43.9, I51.4 | |
| 13 | Myocarditis | B33.2, I40-I41.9, I51.4 | |
| 14 | Alcoholic cardiomyopathy | I42.6 | |
| 15 | Other cardiomyopathy | I42.1-I42.5, I42.7-I42.8, I43-I43.9 | |
| 16 | Atrial fibrillation and flutter | I48-I48.9 | |
| 17 | Aortic aneurysm | I71-I71.9 | |
| 18 | Peripheral artery disease | I70.2-I70.8, I73-I73.9 | |
| 19 | Endocarditis | I33-I33.9, I38-I39.9 | |
| 20 | Other cardiovascular and circulatory diseases | I28-I28.8, I30-I31.1, I31.8-I32.8, I47-I47.9, I51.0-I51.3, I68.0, I72-I72.9, I77-I83.9, I86-I89.0, I89.9, I98, K75.1 | |
| **NEOPLASMS** | |  | |
| **21** | Lip and oral cavity cancer | C00-C08.9, D10.0-D10.5, D11-D11.9 | |
| **22** | Nasopharyx cancer | C11-C11.9, D10.6 | |
| **23** | Other pharynx cancer | C09-C10.9, C12-C13.9, D10.7 | |
| **24** | Esophageal cancer | C15-C15.9, D00.1, D13.0 | |
| **25** | Stomach cancer | C16-C16.9, D00.2, D13.1, D37.1 | |
| **26** | Colon and rectum cancer | C18-C21.9, D01.0-D01.3, D12-D12.9, D37.3-D37.5 | |
| **27** | Liver cancer | C22-C22.9, D13.4 | |
| **28** | Gallbladder and biliary tract cancer | C23-C24.9, D13.5 | |
| **29** | Pancreatic cancer | C25-C25.9, D13.6-D13.7 | |
| **30** | Larynx cancer | C32-C32.9, D02.0, D14.1, D38.0 | |
| **31** | Tracheal, bronchus, and lung cancer | C33-C34.9, D02.1-D02.3, D14.2-D14.3, D38.1 | |
| **32** | Malignant skin melanoma | C43-C43.9, D03-D03.9, D22-D23.9, D48.5 | |
| **33** | Non-melanoma skin cancer | C44-C44.9, D04-D04.9, D49.2 | |
| **34** | Breast cancer | C50-C50.9, D05-D05.9, D24-D24.9, D48.6, D49.3 | |
| **35** | Cervical cancer | C53-C53.9, D06-D06.9, D26.0 | |
| **36** | Uterine cancer | C54-C54.9, D07.0-D07.2, D26.1-D26.9 | |
| **37** | Ovarian cancer | C56-C56.9, D27-D27.9, D39.1 | |
| **38** | Prostate cancer | C61-C61.9, D07.5, D29.1, D40.0 | |
| **39** | Testicular cancer | C62-C62.9, D29.2-D29.8, D40.1-D40.8 | |
| **40** | Kidney cancer | C64-C65.9, D30.0-D30.1, D41.0-D41.1 | |
| **41** | Bladder cancer | C67-C67.9, D09.0, D30.3, D41.4-D41.8, D49.4 | |
| **42** | Brain and central nervous system cancer | C70-C72.9 | |
| **43** | Thyroid cancer | C73-C73.9, D09.3, D09.8, D34-D34.9, D44.0 | |
| **44** | Mesothelioma | C45-C45.9 | |
| **45** | Hodgkin lymphoma | C81-C81.9 | |
| **46** | non-hodgkin lymphoma | C82-C86.6, C96-C96.9 | |
| **47** | multiple myeloma | C88-C90.9 | |
| **48** | leukemia | C91-C95.9 | |
| **49** | Other malignant cancers | C17-C17.9, C30-C31.9, C37-C38.8, C40-C41.9, C47-C4A, C51-C52.9, C57-C57.8, C58-C58.0, C60-C60.9, C63-C63.8, C66-C66.9, C68.0-C68.8, C69-C69.9, C74-C75.8, D07.4, D09.2, D13.2-D13.3, D14.0, D15-D16.9, D28.0-D28.1, D28.7, D29.0, D30.2, D30.4-D30.8, D31-D31.9, D35-D35.2, D35.5-D36, D36.1-D36.7, D37.2, D38.2-D38.5, D39.2, D39.8, D41.2-D41.3, D44.1-D44.8, D48.0-D48.4 | |
| **50** | Other neoplasms | D32-D33.9, D35.3-D35.4, D42-D43.9, D45-D47.9, D49.6, K62.0-K62.1, K63.5, N60-N60.9, N84.0-N84.1, N87-N87.9 | |
| **Chronic respiratory diseases** | | | |
| **51** | Chronic obstructive pulmonary diseases | J41-J44.9 | |
| **52** | Pneumoconiosis | J60-J63.8, J65-J65.0, J92.0 | |
| **53** | Asthma | J45-J46.9 | |
| **54** | Interstitial lung disease and pulmonary sarcoidosis | D86-D86.2, D86.9, J84-J84.9 | |
| **55** | Other chronic respiratory diseases | G47.3, J30-J35.9, J37-J39.9, J66-J68.9, J70, J70.8-J70.9, J82, J91-J92, J92.9 | |
| **Digestive Diseases** | | | |
| **56** | Cirrhosis and other chronic liver diseases | B18-B18.9, I85-I85.9, I98.2, K70-K70.3, K71.7, K74-K74.9, K75.2, K75.4-K76.2, K76.4-K76.9, K77.8 | |
| **57** | Upper digestive system diseases | K21-K21.9, K22.7, K25-K29.9, K31, K31.1-K31.6, K31.8 | |
| **58** | Inguinal, femoral, and abdominal hernia | K40-K42.9, K44-K46.9 | |
| **59** | Inflammatory bowel diseases | K50-K52.9, M09.1 | |
| **60** | Vascular intestinal disorders | K55-K55.9 | |
| **61** | Other digestive diseases | I84-I84.9, K20-K20.9, K22-K22.6, K22.8-K24, K31.0, K31.7, K38-K38.2, K57-K62, K62.2-K62.6, K62.8-K62.9, K64-K64.9, K66.8, K67, K68-K68.9, K77, K90-K90.9, K92.8, K93.8 | |
| **Neurological Disorders** | | | |
| **62** | Alzheimer's disease and other dementias | F00-F03.9, G30-G31.1, G31.8-G31.9 | |
| **63** | Parkinson's disease | G20-G20.9 | |
| **64** | Idiopathic epilepsy | G40-G41.9 | |
| **65** | Multiple sclerosis | G35-G35.9 | |
| **66** | Motor neuron disease | G12.2-G12.9 | |
| **67** | Other neurological disorders | G10-G12.1, G13-G13.8, G23-G24, G24.1-G25.0, G25.2-G25.3, G25.5, G25.8-G26.0, G36-G37.9, G61-G61.9, G70-G72, G72.2-G73.7, G90-G90.9, G95-G95.9, M33-M33.9 | |
| **Mental Disorders** | | | |
| **68** | Eating disorders (anorexia,bulimia) | F50.0-F50.5 | |
| **Substance Use Disorders** | | | |
| **69** | Alcohol use disorders | F10-F10.9, G31.2, G72.1, P04.3, Q86.0, R78.0, X45-X45.9, X65-X65.9, Y15-Y15.9 | |
| **70** | Drug use disorder | F11-F16.9, F18-F19.9, P04.4, P96.1, R78.1-R78.5 | |
| **Diabetes and Kidney Diseases** | | | |
| **71** | DM type 1 | E10-E10.1, E10.3-E10.9, P70.2 | |
| **72** | DM type 2 | E11-E11.1, E11.3-E11.9 | |
| **73** | Chronic Kidney Disease | D63.1, E10.2, E11.2, I12-I13.9, N02-N08.8, N15.0, N18-N18.9, Q61-Q62.8 | |
| **74** | Acute glomerulonephritis | N00-N01.9 | |
| **Skin and subcutaneous Diseases** | | | |
| **75** | Decubitus ulcer | L89-L89.9 | |
| **Musculoskeletal Disorders** | | | |
| **76** | Rheumatoid arthritis | M05-M06.9, M08.0-M08.8 | |
| **77** | Other musculoskeletal disorders | I27.1, I67.7, L93-L93.2, M00-M03.0, M03.2-M03.6, M07-M08, M08.9-M09.0, M09.2-M09.8, M30-M32.9, M34-M36.8, M40-M43.1, M65-M65.0, M71.0-M71.1, M80-M82.8, M86.3-M86.4, M87-M87.0, M88-M89.0, M89.5, M89.7-M89.9 | |
| **Other noncommunicable diseases** | | | |
| **78** | Congenital birth defects | P96.0, Q00-Q07.9, Q10.4-Q18.9, Q20-Q28.9, Q30-Q36, Q37-Q45.9, Q50-Q60.6, Q63-Q86, Q86.1-Q87.8, Q89-Q89.8, Q90-Q93.9, Q95-Q99.8 | |
| **79** | Hemoglobinopathies and hemolytic anemias | D55-D58.9, D59.1, D59.3, D59.5, D60-D61.9, D64.0 | |
| **80** | Neural tube defects | Q00-Q01.9, Q05-Q05.9 | |
| **81** | Congenital heart anomalies | Q20-Q28.9 | |
| **82** | Orofacial clefts | Q35-Q36, Q37-Q37.9 | |
| **83** | Down syndrome | Q90-Q90.9 | |
| **84** | Other chromosomal abnormalities | Q87-Q87.8, Q91-Q93.9, Q95-Q95.9, Q97-Q97.9, Q99-Q99.8 | |
| **85** | Congenital musculoskeletal and limb anomalies | Q65-Q79, Q79.6-Q79.9 | |
| **86** | Urogenital congenital anomalies | P96.0, Q50-Q56.4, Q60-Q60.6, Q63-Q64.9 | |
| **87** | Digestive congenital anomalies | Q38-Q45.9, Q79.0-Q79.5 | |
| **88** | Other congenital birth defects | Q02-Q04.9, Q06-Q07.9, Q10.4-Q18.9, Q30-Q34.9, Q57, Q80-Q86, Q86.1-Q86.8, Q89-Q89.8 | |
| **89** | Polycystic ovarian syndrome | E28.2 | |
| **90** | Endometriosis | N80-N80.9 | |
| **91** | Genital prolapse | N81-N81.9 | |
| **92** | Thalassemias | D56-D56.9 | |
| **93** | Sickle cell disorders | D57-D57.8 | |
| **94** | G6PD deficiency | D55-D55.2 | |
| **95** | Other hemoglobinopathies and hemolytic anemias | D55.3-D55.9, D58-D58.9, D59.1, D59.3, D59.5, D60-D61.9, D64.0 | |
| **96** | Endocrine, metabolic, blood, and immune disorders | D52.1, D59.0, D59.2, D59.6, D66-D67, D68.0-D69.8, D70-D75.8, D76-D78.8, D86.8, D89-D89.3, E03-E07.1, E09-E09.9, E15.0, E16.0-E16.9, E20-E28.1, E28.3-E34.8, E36-E36.8, E65-E68, E70-E85.2, E88-E89.9, G24.0, G25.1, G25.4, G25.6-G25.7, G72.0, G93.7, G97-G97.9, I95.2-I95.3, I97-I97.9, I98.9, J70.0-J70.5, J95-J95.9, K43-K43.9, K62.7, K91-K91.9, K94-K95.8, M87.1, N14-N14.4, N65-N65.1, N99-N99.9, P96.2, P96.5, R50.2 | |

## Section 3. List of regions

The five regions represent the following provinces in Indonesia: Banten, DKI Jakarta, West Java, Central Java, Yogyakarta and East Java (Region 1), West Sumatera, Riau, South Sumatera, Lampung, Bali and West Nusa Tenggara (Region 2), Aceh, North Sumatera, Jambi, Bengkulu, Bangka Belitung, Riau Island, West Kalimantan, North Sulawesi, Central Sulawesi, Southeast Sulawesi, West Sulawesi, South Sulawesi and Gorontalo (Region 3), South Kalimantan, East Kalimantan, North Kalimantan and Central Kalimantan (Region 4) and East Nusa Tenggara, Maluku, North Maluku, Papua and West Papua (Region 5)

## Section 4. Supplementary results

Figure S1 Districts caterpillar plot for out- and inpatient costs

**Figure S1.** District caterpillar plots for out- and inpatient costs showing the residuals from all 514 districts in the sample with 95% confidence intervals. The residuals show the starting point of the costs for in- and outpatient costs. The graph reveals that for a substantial number of districts, the 95% CI does not overlap with the horizontal line at zero, indicating that the in- and outpatient costs related to chronic diagnoses among adults under National health insurance (NHI) are significantly above (above the zero line) or below average (below the zero line).

**Table S2.** Characteristics of the Healthcare System at the baseline year based on regions

| **District's characteristics** | **Region** | | | | | **Overall** | **p-value** |
| --- | --- | --- | --- | --- | --- | --- | --- |
|  | **Region 1** | **Region 2** | **Region 3** | **Region 4** | **Region 5** |  |  |
| Number of Districts | 119 (23.2) | 82 (16.0) | 186 (36.2) | 42 (8.2) | 85 (16.5) | 514 (100.0) | <0.001 |
| Density PHC per 10,000 NHI members | 1.18 (0.50) | 1.70 (0.77) | 1.95 (1.66) | 2.52 (0.76) | 2.27 (1.37) | 1.83 (1.29) | <0.001 |
| Density hospitals per 10,000 NHI members | 0.39 (0.38) | 0.42 (0.41) | 0.35 (0.32) | 0.47 (0.23) | 0.21 (0.22) | 0.35 (0.34) | <0.001 |
| Density PHC per 10,000 population in the district | 0.77 (0.39) | 0.96 (0.44) | 1.19 (0.56) | 1.36 (0.36) | 1.77 (0.92) | 1.17 (0.66) | <0.001 |
| Density hospitals per 10,000 population in the district | 0.26 (0.27) | 0.26 (0.32) | 0.23 (0.20) | 0.25 (0.14) | 0.17 (0.17) | 0.23 (0.23) | <0.001 |
| Proportion (%) of NHI members who ever used NHI services | 0.43 (0.04) | 0.42 (0.05) | 0.38 (0.05) | 0.37 (0.05) | 0.30 (0.08) | 0.38 (0.07) | <0.001 |
| Fiscal Capacity of districts |  |  |  |  |  |  |  |
| Very Low | 80 (67.2) | 35 (42.7) | 80 (43.0) | 0 (0.0) | 32 (37.6) | 227 (44.2) | <0.001 |
| Low | 17 (14.3) | 23 (28.0) | 68 (36.6) | 0 (0.0) | 22 (25.9) | 130 (25.3) |  |
| High | 12 (10.1) | 16 (19.5) | 31 (16.7) | 16 (38.1) | 22 (25.9) | 97 (18.9) |  |
| Very High | 10 (8.4) | 8 (9.8) | 7 (3.8) | 26 (61.9) | 9 (10.6) | 60 (11.7) |  |

Primary Healthcare (PHC) is the number of clinics, puskesmas and general practitioners contracted under the National Health Insurance Programme. The baseline year was defined as the first enrolment of the individual-level variable that contributes to the development of the district-level variable (proportion of NHI members who used NHI services). The names of the districts in each region are listed in the online supplementary methods. PHC: primary healthcare. Statistics are presented as mean (SD) for the continuous variables density of PHC, density of hospitals and proportion of NHI members who ever used NHI services, and N (%) for the categorical variables number of districts and fiscal capacity of districts. P-values for continuous variables were derived from analysis of variance (ANOVA), and p-values for categorical variables (number of districts and district fiscal capacity) were derived from a chi-squared test (χ2).

## Multilevel analysis of null models

The first null model was a single-level model, the second null model included random effects at the district level, and the third null model included random effects at the district and households levels (Table S3). The likelihood ratio test was significant for two-level and three-level models, showing that multilevel analysis was more robust than the single-level analysis. Furthermore, these findings also showed that the three-level model was a better fit for the analysis. For outcomes related to outpatient costs, Intraclass correlation (ICC) in the null model (Model 3) was 3.0% and 38% at the district and household levels, respectively (Table S3). Intriguingly, the ICC for inpatient costs were similar to that for outpatient costs, i.e. 3% and 26% at the district and household levels, respectively. The ICC for both out- and inpatient costs indicated that variable at the individual level explained most of the variation, followed by household and district level. At the household level, less variation in inpatient costs was found compared to outpatient costs.

**Table S3.** Multilevel analysis of null models with the transformed outcome: individual-level model (Model 1), district effects (Model 2), as well as districts and households effects (Model 3).

| **Variable** | **Outpatient Cost** | | | **Inpatient Cost** | | |
| --- | --- | --- | --- | --- | --- | --- |
|  | **Model 1**  **(95% CI)** | **Model 2**  **(95% CI)** | **Model 3**  **(95% CI)** | **Model 1**  **(95% CI)** | **Model 2**  **(95% CI)** | **Model 3**  **(95% CI)** |
| Constant | 4.66  (4.65, 4.66) | 4.51  (4.49, 4.53) | 4.44  (4.43, 4.47) | 6.71  (6.71, 6.72) | 6.66  (6.65, 6.67) | 6.66  (6.64, 6.67) |
| Between district variance | - | 0.23  (0.22, 0.25) | 0.21  (0.19,0.22) | - | 0.14  (0.13, 0.15) | 0.13  (0.12, 0.14) |
| Between household variance | - | - | 0.76  (0.76, 077) | - | - | 0.39  (0.39, 0.40) |
| ICC (district) | - | 0.03  (0.03, 0.04) | 0.03  (0.02,0.03) | - | 0.03  (0.02,0.03) | 0.03  (0.02,0.03) |
| ICC (household) | - | - | 0.39  (0.38, 0.39) | - | - | 0.26  (0.25,0.27) |
| Group level | individuals | district, individuals | district, household, individuals | individuals | district, individuals | district, household, individuals |
| Number of groups | 215,660 individuals | 514 districts,  215,660 individuals | 514 districts,  131,839 households,  215,660 individuals | 114,918 individuals | 514 districts,  114,918 individuals | 514 districts,  91,009 households,  114,918 individuals |
| Likelihood ratio test (LR) | - | 6,622.25 | 25,893.71 | - | 2,637.30 | 4,639.33 |
| Prob>chi2 | - | 0.0000 | 0.0000 | - | 0.0000 | 0.0000 |

Model 1: Single-level model (individual level), Model 2: Two-level model (individual, district), Model 3: Three-level model (individual, household, district). Intraclass correlation (ICC) measures the degree of homogeneity in costs of care for out- and inpatients within a household and within a district. The likelihood ratio test is significant for two- and three-level models showing that multilevel analysis is more robust and three-level models are a better fit for the analysis. The outcome is transformed using an inversed hyperbolic sine transformation (sinh ^-1^, IHS).

Figure S2. Interaction analysis between group of diagnosis and household subsidized status on Predicted Outpatient Costs

Figure S3. Interaction analysis between group of diagnosis and household subsidized status on predicted inpatient costs*.*

**Table S4.** Characteristics of the study participants at the baseline year based on households subsidized status: A sensitivity analysis*

| **Variables** | **Households subsidy status** | | **Total** |
| --- | --- | --- | --- |
|  | **Non-subsidized** | **Subsidized** |  |
| **Individual level** |  |  |  |
| **Overall** | 153,534 (56.6) | 117,531 (43.4) | 271,065 (100.0) |
| **Disease groups** |  |  |  |
| (1) No CVDs, but with single chronic morbidity | 107,567 (70.1) | 93,224 (79.3) | 200,791 (74.1) |
| (2) No CVDs, but with multimorbidity | 20,427 (13.3) | 12,158 (10.3) | 32,585 (12.0) |
| (3) CVDs, but no comorbidity | 10,657 (6.9) | 6,580 (5.6) | 17,238 (6.4) |
| (4) CVDs and one comorbidity | 8,121 (5.3) | 3,661 (3.1) | 11,782 (4.3) |
| (5) CVDs and multimorbidity | 6,762 (4.4) | 1,908 (1.7) | 8,669 (3.2) |
| **Sex** |  |  |  |
| Men | 67,322 (43.9) | 45,785 (39.0) | 113,107 (41.7) |
| Women | 86,212 (56.1) | 71,746 (61.0) | 157,958 (58.3) |
| **Age group** |  |  |  |
| 30-39 | 38,832 (25.3) | 22,491 (19.1) | 61,323 (22.6) |
| 40-49 | 37,142 (24.2) | 28,910 (24.6) | 66,052 (24.4) |
| 50-59 | 38,102 (24.8) | 29,250 (24.9) | 67,352 (24.8) |
| 60-69 | 22,873 (14.9) | 19,297 (16.4) | 42,170 (15.6) |
| 70-79 | 12,510 (8.2) | 13,155 (11.2) | 25,665 (9.5) |
| >=80 | 4,075 (2.6) | 4,428 (3.8) | 8,503 (3.1) |
| **Marital status** |  |  |  |
| Not married | 11,809 (7.7) | 8,478 (7.2) | 20,287 (7.5) |
| Married | 128,853 (84.0) | 30,527 (25.9) | 159,380 (58.8) |
| Divorced | 12,170 (7.9) | 3,536 (3.1) | 15,706 (5.8) |
| Undefined | 702 (0.4) | 74,990 (63.8) | 75,692 (27.9) |
| **Region** |  |  |  |
| Region 1 | 92,937 (60.5) | 72,986 (62.1) | 165,923 (61.2) |
| Region 2 | 20,272 (13.2) | 15,100 (12.9) | 35,372 (13.0) |
| Region 3 | 29,111 (19.0) | 23,886 (20.3) | 52,997 (19.6) |
| Region 4 | 7,069 (4.6) | 2,753 (2.3) | 9,822 (3.6) |
| Region 5 | 4,145 (2.7) | 2,806 (2.4) | 6,951 (2.6) |
|  |  |  |  |
| **Household level** |  |  |  |
| **Overall** | 153,280 (68.7) | 69,877 (31.3) | 223,157 (100.0) |
| Proportions (%) of household members with multimorbidity (SD) | 21.87 (31.54) | 14.84 (25.25) | 21.96 (31.64) |
| Mean total household number (SD) | 1.94 (0.59) | 1.48 (0.25) | 2.21 (0.87) |

Data is presented as N (number of observations), mean (SD) and the proportion (%) based on the households category (Non-subsidized vs subsidized). The baseline year is the year when the participants first enrolled (first diagnosed in the dataset) in the cohort. CVDs - cardiovascular diseases. All analyses were weighted with analytical sample weight. *A sensitivity analysis: households with both subsidized and non-subsidized members were categorized as non-subsidized households

Table S5. Average of annual outpatient and inpatient costs related to the presence of CVDs with or without other chronic disease comorbidities in US dollars between 2016 and 2018: A sensitivity analysis*

| **Variable** | **Outpatient costs in USD**  **Mean (SD)** | | | **Inpatient costs in USD**  **Mean (SD)** | | |
| --- | --- | --- | --- | --- | --- | --- |
|  | **Overall**  **(N=215,660)** | **Individuals who belonged to non-subsidized households**  **(N=173,820)** | **Individuals who belonged to subsidized households**  **(N=41,840)** | **Overall**  **(N=114,918)** | **Individuals who belonged to non -subsidized households**  **(N=84,642)** | **Individuals who belonged to subsidized households**  **(N=30,276)** |
| **Overall** | 179.6 (703.5) | 195.9 (748.9) | 135.7 (560.2) | 634.8 (940.9) | 719.6 (1054.7) | 468.4 (631.3) |
| **Groups of patients** |  |  |  |  |  |  |
| (1) No CVDs, but with single chronic morbidity | 121.8 (500.9) | 127.0 ( 523.6) | 109.3 (441.7) | 441.6 (508.9) | 480.3 (552.4) | 376.7 (418.4) |
| (2) No CVDs, but with multimorbidity | 256.6 (964.1) | 283.5 (1041.9) | 176.8 (676.7) | 757.1 (945.9) | 848.5 (1048.5) | 561.5 (633.3) |
| (3) CVDs, but no comorbidity | 107.1 (176.5) | 117.8 (190.3) | 79.3 (130.6) | 610.7 (950.0) | 717.0 (1120.4) | 441.8 (543.7) |
| (4) CVDs and one comorbidity | 214.2 (702.45) | 225.5 (700.5) | 177.0 (707.8) | 750.2 (1162.3) | 841.2 (1273.1) | 534.2 (802.8) |
| (5) CVDs and multimorbidity | 415.2 (1214.12) | 438.3 (1236.2) | 311.6 (1104.6) | 1135.2 (1602.9) | 1214.2 (1672.7) | 846.5 (1276.3) |

Costs associated with out- and inpatient visits were presented in the U.S. dollar (USD) value for 2018. In- and outpatient costs were the average total costs associated with chronic diagnosis per patient annually. All costs presented in the analysis used a payer’s perspective based on the tariff paid by the healthcare insurance agency [25]. CVDs - cardiovascular diseases. All analyses were weighted with analytical sample weight. *A sensitivity analysis: households with both subsidized and non-subsidized members were categorized as non-subsidized households **Table S6.** Association of individual, household, and district level characteristics with outpatient healthcare costs related to the presence of CVDs with or without other chronic disease comorbidities : A sensitivity analysis*

| **Variable** | **Outpatient costs** | | | | | |
| --- | --- | --- | --- | --- | --- | --- |
|  | **Model 1 ^a^**  **coefficient** (n=215,660) | **(95% CI)** | **Model 2 ^b^**  **coefficient** (n=215,660) | **(95% CI)** | **Model 3 ^c^**  **coefficient** (n=215,660) | **(95% CI)** |
| **Individual level** |  |  |  |  |  |  |
| **Group** |  |  |  |  |  |  |
| (1) No CVDs, but with single chronic morbidity | REF | REF | REF | REF | REF | REF |
| (2) No CVDs, but with multimorbidity | 0.55*** | (0.54, 0.56) | 0.49*** | (0.47, 0.51) | 0.49*** | (0.48,0.51) |
| (3) CVDs, but no comorbidity | 0.20*** | (0.18, 0.23) | 0.17*** | (0.14, 0.19) | 0.17*** | (0.14,0.19) |
| (4) CVDs and one comorbidity | 0.56*** | (0.53, 0.58) | 0.49*** | (0.47, 0.51) | 0.49*** | (0.47,0.51) |
| (5) CVDs and multimorbidity | 1.07*** | (1.05, 1.09) | 1.00*** | (0.98, 1.02) | 1.00*** | (0.98,1.02) |
| **Household type** |  |  |  |  |  |  |
| Non-subsidized | REF | REF | REF | REF | REF | REF |
| Subsidized | -0.08*** | (-0.11, -0.06) | -0.08*** | (-0.11, -0.06) | -0.08*** | (-0.11,-0.06) |
| **Group*Household type** |  |  |  |  |  |  |
| (1)*Subsidized |  |  |  |  |  |  |
| (2)*Subsidized | -0.16*** | (-0.20, -0.13) | -0.16*** | (-0.20, -0.13) | -0.16*** | (-0.19,-0.13) |
| (3)*Subsidized | -0.14*** | (-0.19, -0.09) | -0.13*** | (-0.18, -0.08) | -0.14*** | (-0.18,-0.09) |
| (4)*Subsidized | -0.17*** | (-0.22, -0.12) | -0.16*** | (-0.21, -0.12) | -0.16*** | (-0.21,-0.12) |
| (5)*Subsidized | -0.24*** | (-0.29, -0.19) | -0.24*** | (-0.29, -0.18) | -0.24*** | (-0.29,-0.19) |
| **Household level** |  |  |  |  |  |  |
| Proportions of household members with multimorbidity | - | - | 0.16*** | (0.14, 0.18) | 0.16*** | (0.15, 0.19) |
| The mean number of household members | - | - | 0.01*** | (0.01, 0.02) | 0.02*** | (0.01, 0.03) |
| **District level** |  |  |  |  |  |  |
| Proportion of primary care per 10000 | - | - | - | - | 0.02** | (0.00, 0.03) |
| Proportion of hospitals per 10000 | - | - | - | - | 0.06*** | (0.02, 0.11) |
| % of NHI members who utilized healthcare | - | - | - | - | -0.01 | (-0.15, 0.12) |
| Fiscal category |  |  |  |  |  |  |
| Low |  |  |  |  | 0.01 | (-0.01, 0.02) |
| Middle | - | - | - | - | -0.01 | (-0.02, 0.01) |
| High | - | - | - | - | -0.03 | (-0.05, -0.01) |
| Very high | - | - | - | - |  |  |
| Intercept | 4.51 | (4.47, 4.55) | 4.45 | (4.40, 4.49) | 4.41 | (4.34, 4.49) |
| District level’s variance | 0.16 | (0.15, 0.17) | 0.16 | (0.15, 0.17) | 0.16 | (0.15, 0.18) |
| Household level’s variance | 0.69 | (0.68, 0.69) | 0.68 | (0.68, 0.69) | 0.69 | (0.68, 0.69) |
| ICC (district level) | 0.02 | (0.01, 0.02) | 0.02 | (0.01, 0.02) | 0.02 | (0.01, 0.02) |
| ICC (household level) | 0.35 | (0.34, 0.35) | 0.35 | (0.34, 0.35) | 0.35 | (0.34, 0.35) |
| Likelihood ratio test (LR) | 18,633.45 |  | 18,429.38 |  | 17,476.95 |  |

Coefficient is transformed using an inversed hyperbolic sine transformation (sinh ^-1^, IHS). We retransformed β coefficients to U.S. dollar (USD) using cost value (x)=(exp^2x-1^)/(2exp^x^). P-values were statistically significant at 1 percent (***), 5 percent (**) or 10 percent (*).

^a^ Model 1: Multilevel linear regression with transformed outcome, cross-level between individual and controlled for individual-level covariates.

^b^ Model 2: Multilevel linear regression with transformed outcome and controlled for individual and household-level covariates.

^c^ Model 3: Multilevel linear regression with transformed outcome and controlled for individual, household, and district-level covariates.

All models were also adjusted for sex, age, marital status, type of primary healthcare centres registered, and regions. *A sensitivity analysis: households with both subsidized and non-subsidized members were categorized as non-subsidized households

**Table S7.** Association of individual, household, and district level characteristics with inpatient healthcare costs related to the presence of CVDs with or without other chronic disease comorbidities: A sensitivity analysis*

| **Variable** | **Inpatient costs** | | | | | |
| --- | --- | --- | --- | --- | --- | --- |
|  | **Model 1 ^a^**  **Coefficient**  (n=114,918) | **(95% CI)** | **Model 2 ^b^**  **Coefficient** (n=114,918) | **(95% CI)** | **Model 3 ^c^**  **Coefficient** (n=114,918) | **(95% CI)** |
| **Individual level** |  |  |  |  |  |  |
| **Group** |  |  |  |  |  |  |
| (1) No CVDs, but with single chronic morbidity | REF | REF | REF | REF | REF | REF |
| (2) No CVDs, but with multimorbidity | 0.39*** | (0.38, 0.40) | 0.38*** | (0.36, 0.39) | 0.38*** | (0.36, 0.39) |
| (3) CVDs, but no comorbidity. | 0.18*** | (0.17, 0.20) | 0.18*** | (0.16, 0.19) | 0.18*** | (0.16, 0.20) |
| (4) CVDs and one comorbidity | 0.34*** | (0.33, 0.36) | 0.33*** | (0.31, 0.35) | 0.33*** | (0.31, 0.35) |
| (5) CVDs and multimorbidity | 0.64*** | (0.62, 0.65) | 0.62*** | (0.60, 0.64) | 0.62*** | (0.61, 0.64) |
| **Household type** |  |  |  |  |  |  |
| Non-subsidized | REF | REF | REF | REF | REF | REF |
| Subsidized | -0.21*** | (-0.23,-0.19) | -0.21*** | (-0.23,-0,19) | -0.21*** | (-0.23, -0.18) |
| **Group*Household type** |  |  |  |  |  |  |
| (1)*Subsidized |  |  |  |  |  |  |
| (2)*Subsidized | -0.08*** | (-0.11, -0.05) | -0.08*** | (-0.10, -0,05) | -0.07*** | (-0.10, -0.05) |
| (3)*Subsidized | -0.09*** | (-0.12, -0.06) | -0.09*** | (-0.12, -0.06) | -0.09*** | (-0.13, -0.06) |
| (4)*Subsidized | -0.12*** | (-0.15, -0.08) | -0.11*** | (-0.15, -0.08) | -0.12*** | (-0.15, -0.08) |
| (5)*Subsidized | -0.12*** | (-0.15, -0.07) | -0.12*** | (-0.15, -0.08) | -0.11*** | (-0.15, -0.07) |
| **Household level** |  |  |  |  |  |  |
| Proportions of household members with multimorbidity | - | - | 0.03*** | (0.02, 0.05) | 0.04*** | (0.03, 0.05) |
| The mean number of household members | - | - | -0.01*** | (-0.02,-0.04) | -0.01** | (-0.02, -0.004) |
| **District level** |  |  |  |  |  |  |
| Proportion of primary care per 10,000 | - | - | - | - | 0.04*** | (0.03, 0.05) |
| Proportion of hospitals per 10,000 | - | - | - | - | -0.01 | (-0.04, 0.02) |
| % of NHI members who utilized healthcare | - | - | - | - | -0.00 | (-0.12, 0.11) |
| Fiscal category |  |  |  |  |  |  |
| Low |  |  |  |  |  |  |
| Middle | - | - | - | - | -0.03*** | (-0.05, -0.02) |
| High | - | - | - | - | -0.07*** | (-0.09, -0.06) |
| Very high | - | - | - | - | -0.11*** | (-0.13, -0.08) |
| Intercept | 6.70 | (6.67, 6.73) | 6.72 | (6.68, 6.74) | 6.72 | (6.66, 6.78) |
| District level’s variance | 0.09 | (0.09, 0.11) | 0.09 | (0.09, 0.11) | 0.11 | (0.10, 0.12) |
| Household level’s variance | 0.34 | (0.33, 0.35) | 0.34 | (0.33, 0.35) | 0.33 | (0.33, 0.35) |
| ICC (district level) | 0.02 | (0.01, 0.02) | 0.02 | (0.01, 0.02) | 0.02 | (0.02, 0.03) |
| ICC (household level) | 0.21 | (0.19, 0.22) | 0.21 | (0.20, 0.22) | 0.21 | (0.20, 0.22) |
| Likelihood ratio test (LR) | 2,730.05 |  | 2,714.64 |  | 2,713.98 |  |

Coefficient is transformed using an inversed hyperbolic sine transformation (sinh ^-1^, IHS). To retransform β coefficient to U.S. dollar (USD) cost value (x)=(exp^2x-1^)/(2exp^x^). P-value statistically significant at 1 percent (***), 5 percent (**) or 10 percent (*) of the confidence intervals.

^a^ Model 1 Multilevel linear regression with transform outcome, cross-level between individual and controls for individual-level covariates.

^b^ Model 2 Multilevel linear regression with transform outcome and controls for individual and household covariates

^c^ Model3 Multilevel linear regression with transform outcome and controls for individual, household, and district covariates

All models adjusted with sex, age, marital status, type of primary healthcare centres registered, and regions. *A sensitivity analysis: households with both subsidized and non-subsidized members were categorized as non-subsidized households

**Figure S4.** Predicted mean annual healthcare costs per patient by diagnosis group (2016-2018) in U.S. dollars (USD): A sensitivity analysis*

Costs associated with out- and inpatient visits are presented in the USD value for 2018. Predicted costs were calculated from the transformed coefficient to USD cost value (x)=(exp^2x-1^)/(2exp^x^). Costs were predicted using three-level multilevel linear regression with transform outcome and controlled for individual, household, and district covariates. *A sensitivity analysis: households with both subsidized and non-subsidized members were categorized as non-subsidized households
